# Dynamics of Dual Prism Adaptation: Relating Novel Experimental Results to a Minimalistic Neural Model

**DOI:** 10.1371/journal.pone.0076601

**Published:** 2013-10-29

**Authors:** Orlando Arévalo, Mona A. Bornschlegl, Sven Eberhardt, Udo Ernst, Klaus Pawelzik, Manfred Fahle

**Affiliations:** 1 Institute for Theoretical Physics, University of Bremen, Bremen, Germany; 2 Institute for Human Neurobiology, University of Bremen, Bremen, Germany; 3 Henry Wellcome Laboratories for Vision Sciences, City University London, England, United Kingdom; National Institute of Mental Health, United States of America

## Abstract

In everyday life, humans interact with a dynamic environment often requiring rapid adaptation of visual perception and motor control. In particular, new visuo–motor mappings must be learned while old skills have to be kept, such that after adaptation, subjects may be able to quickly change between two different modes of generating movements (‘dual–adaptation’). A fundamental question is how the adaptation schedule determines the acquisition speed of new skills. Given a fixed number of movements in two different environments, will dual–adaptation be faster if switches (‘phase changes’) between the environments occur more frequently? We investigated the dynamics of dual–adaptation under different training schedules in a virtual pointing experiment. Surprisingly, we found that acquisition speed of dual visuo–motor mappings in a pointing task is largely independent of the number of phase changes. Next, we studied the neuronal mechanisms underlying this result and other key phenomena of dual–adaptation by relating model simulations to experimental data. We propose a simple and yet biologically plausible neural model consisting of a spatial mapping from an input layer to a pointing angle which is subjected to a global gain modulation. Adaptation is performed by reinforcement learning on the model parameters. Despite its simplicity, the model provides a unifying account for a broad range of experimental data: It quantitatively reproduced the learning rates in dual–adaptation experiments for both direct effect, i.e. adaptation to prisms, and aftereffect, i.e. behavior after removal of prisms, and their independence on the number of phase changes. Several other phenomena, e.g. initial pointing errors that are far smaller than the induced optical shift, were also captured. Moreover, the underlying mechanisms, a local adaptation of a spatial mapping and a global adaptation of a gain factor, explained asymmetric spatial transfer and generalization of prism adaptation, as observed in other experiments.

## Introduction

Visuo–motor mappings are efficiently learned, adapted and re–adapted throughout life. In many cases, adaptation takes place in a matter of few trials and on a timescale of minutes. Prolonged adaptation leads to an acquisition of new skills, and subjects become capable to switch rapidly between various learned visuo–motor mappings [1]–[3]. Adaptation and learning are often non–local, such that new skills generalize to situations differing from the particular situations used for training [4]–[6].

A daily situation exemplifies these observations: Learning to ride a bike which differs from the one you already master. Being subjected to a suddenly new or distorted situation normally causes the experience that an action just performed is not adequate (the *direct effect*) and needs to be corrected or adapted. If after successful adaptation, the normal non–distorted situation is reestablished, the subject usually experiences the necessity to re–adapt to the original condition (the *aftereffect*). A fundamental question is whether it matters for learning to master both bikes (*dual*–*adaptation*) how often the training switches between bikes? A second important aspect concerns the generalization: having mastered the new bike, will I be able to master it equally well when adding heavy saddlebags?

Dual prism adaptation provides a suitable paradigm to study the processes that occur during visuo–motor learning: Subjects are instructed to perform pointing movements towards a visual target. After some familiarization movements, the subjects have to wear glasses which induce a relatively uniform horizontal shift of the visual world, either leftwards or rightwards. When first confronted with such a visual shift, observers have the strong tendency for ballistic movements to deviate leftwards or rightwards (depending on the type of prisms) of the correct reaching trajectory towards a target. This behavior results in an error between final hand and target positions, the *direct effect*. This error is subsequently corrected by observers during further pointing movements, until they acquire a new mapping between proprioceptive and visual space. A pointing error in the opposite direction, the *aftereffect*, is observed when returning from the shifted to the normal condition, i.e. when removing the glasses. Similar effects are also seen when observers are confronted with force fields during hand movements [7]. The stepwise reduction of both direct effect and aftereffect by multiple changes between phases with the shifted and non–shifted viewing condition represents a measure of *dual*–*adaptation*, i.e. dual visuo–motor mapping learning. Although in modern experimental setups, and in the studies reviewed in this article, visual shifts are realized by a virtual reality setup on specialized computer hardware, for historical reasons we will refer to the main paradigm as ‘dual prism adaptation’.

Humans acquire and implement multi–modal visuo–motor mappings such that they are able to switch very quickly from one motor control pattern to another [1]–[3]. If a cue is available indicating which situation is present, this switching becomes practically instantaneous after prolonged training. According to previous studies, adaptation takes place mostly in the proprioception, but also in vision [8], [9]. Pointing while looking through prisms is a task differing from riding a new bike, but in both situations the coding and control of angles of our extremities by the proprioceptive system is fundamental.

Here we investigate the dynamics of dual–adaptation in a combination of experiments and modelling. In particular, we test two hypotheses about putative factors determining the speed of dual prism adaptation: the number of phase changes and the total number of movements.

- The first hypothesis is that dual mappings are learnt faster the more often a subject experiences a *phase change* (i.e. prisms off versus prisms on, and prisms off versus prisms on). Then dual–adaptation should depend mainly on the number of changes between prism conditions.
- The alternative hypothesis is that the acquisition of multiple mappings is mainly regulated by the number of trials with an effective feedback, e.g. with a pointing error. This would mean that dual–adaptation should mainly depend on the total number of executed movements which provide feedback about the error, but not on the number of phase changes.

In this contribution, we will show that to a first approximation, only the number of executed movements determines the speed of dual–adaptation, if these movements are performed on a tight temporal schedule in one experimental session. The impact of the number of phase changes was very small, as well as the size of the visual shift.

These new results impose strong constraints on possible models and mechanisms explaining dual–adaptation. The most characteristic features of prism adaptation [8], [10]–[12] and of dual prism adaptation [2]–[4] have already been identified and discussed in detail. Different mechanisms, independently responsible for subsets of related motor adaptation features, have been proposed. The approaches comprise two main groups: The first group consists of complex feedback adaptive controllers based on internal models for motor control and trajectory planning, or on learnable basis functions [5], [13]–[15]. The second group comprises probabilistic and statistical frameworks such as Bayesian inference, optimum information integration and error estimation via Kalman filters [16]–[18].

However, approaches on neural models for dual–adaptation, as to be found in [19], more inspired by and focused on putative biological implementations, are less explored. More importantly, none of the above mentioned abstract conceptualizations reproduces in a unified manner the important features of dual–adaptation. The importance of a neural approach is its potential to identify the ‘machinery’ behind human adaptation and learning. It allows for understanding of and predictions about capabilities and limitations of the human visuo–motor system.

In the modelling part of our study, we will show that a minimalistic neural network is capable of reproducing our experimental results on the learning dynamics during dual–adaptation both qualitatively and quantitatively. In addition, and despite its structural simplicity, our approach provides a unifying account for other phenomena characteristic for prism adaptation, such as the generalization of adaptation to different target positions. Thus, the model sheds light on the mechanisms underlying the experimentally observed phenomena.

The proposed model uses two forms of inputs: The first input is used to estimate the target location from a neuronal representation of visual space in the early visual areas. The second input informs about the situation (condition) that the subject currently faces. It contains a cognitive component of the paradigm by modeling the abstract realization that ‘something has changed’, which is necessary for a subject to choose the appropriate motor control pattern actually needed for the current situation.

We show that our model can reproduce quantitatively key features of experimental data, as well as predict motor behavior when certain experimental conditions are manipulated. The constitution of this model is based on biologically plausible mechanisms, e.g. the coding of angles of eyes and other body parts by muscle spindles [20], and on behavioral responses, e.g. realignment of proprioception and vision [3], [21]. With a plausible learning mechanism inspired by reinforcement learning [22], our model finds a configuration of its internal parameters (synaptic weights) that allows for acquisition and storage of dual sensory–motor mappings.

This paper is organized in four main sections. The next section contains a description of the experimental setup and paradigm for studying dual–adaptation, together with a short description of methods used for data analysis, and a full description of the model. The second section reports the main experimental findings. The third section compares the results obtained from experiments and from model simulations. The final section discusses the results and presents testable validations and refinements of the model. The appendix S1 holds additional information for the model setup and generalization of results.

## Methods

### Ethics Statement

All experiments were approved of by the Bremen University ethics committee. Prior to the experiment, subjects were briefed about the experimental procedure and gave their written informed consent. The guidelines in the declaration of Helsinki (2008) were strictly followed throughout the experiment.

### Participants

The 50 subjects (21 male) were right–handed volunteers, mostly students at Bremen University, aged 20 to 30 years (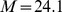, 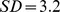). All subjects had normal, or corrected-to-normal visual acuity (Snellen: 20/20) and ‘normal’ stereoscopic vision (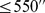), they were also naive to prism addaptation. Subjects were paid 8 EUR per hour for their participation.

### Experimental setup and task

**Figure 1:**
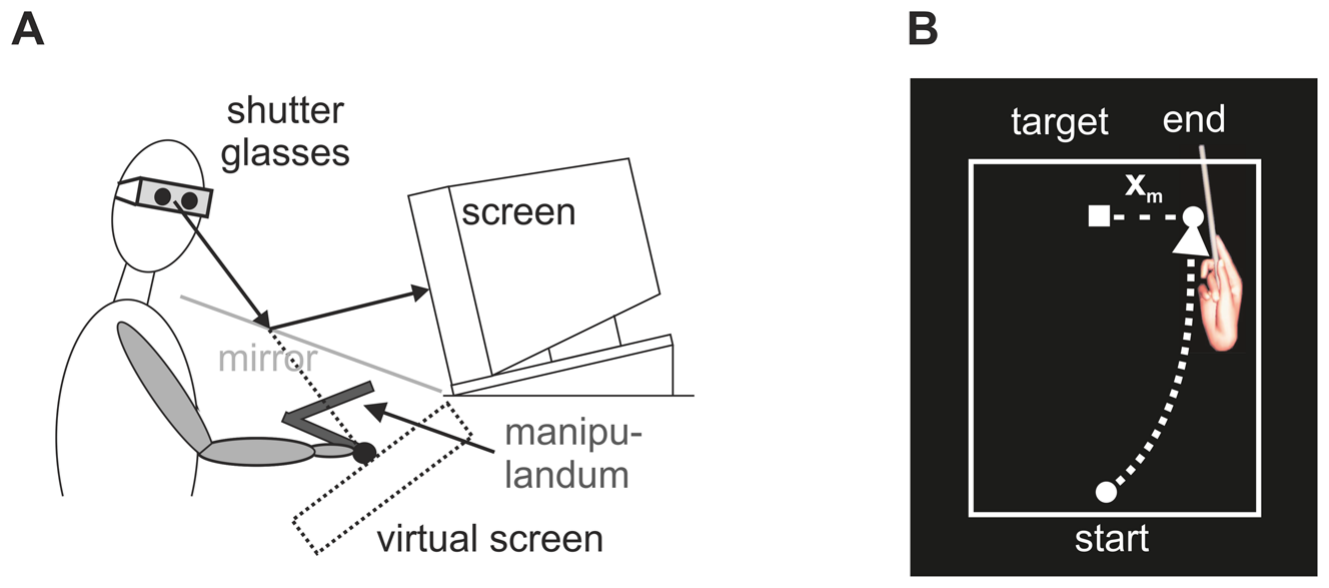
Virtual reality setup. **A**) A Subject sees the VR screen via a mirror, and executes pointing movements with a manipulandum. A head post constrains head movements of the subject, while wearing shutter glasses for 3D perception. **B**) VR screen showing the virtual hand, the target, and the frame.

In the experiment we used a virtual reality (VR) setup with a CRT screen viewed via a mirror (figure 1). In the setup the subject was looking down at the working area hidden behind the mirror. This hidden working area (approx. size 380 mm width×260 mm height×190 mm depth) was designed to coincide with natural hand movement space. Hand movements in the working area were recorded in six degrees of freedom (3 spatial, 3 rotational) with a manipulandum (Phantom Premium 1.5 HF) and used in real time for controlling the position and orientation of a virtual hand in the VR. Subjects held the manipulandum handle in their right hand with the index finger parallel to the handle and oriented towards the virtual screen. A box with a button mounted on its top was used as starting position for all movements. Shutter glasses (Crystal Edge) were used to create a 3D visual VR adjusted to the subjects' individual pupillary distance. The virtual screen was realized as a reflection of the real screen on the mirror, and appeared perpendicular to the viewing axis 47 cm in front of the subject. On the screen a target with a surrounding rectangular frame (165 mm height×250 mm width) and a virtual hand could be displayed. A white rectangle was used as the target, shown 6 cm behind the surface of the virtual screen. The virtual hand was realized by a realistic 3D model of a hand holding a stick, shown in a pointing posture matching the real hand holding the manipulandum handle in the hidden working area. This 3D device is essential for a realistic feeling in the VR and for investigating adaptation. Without a realistic feedback, only corrections based on the consciously perceived pointing error might occur. The rectangular frame was shown at zero disparity horizontally centered around the target for allowing easy and successful fusion in the VR.

The task for the subjects was to reach towards the target with a pointing movement of the virtual hand under different viewing conditions. The target was displayed on the screen, either on the right side (original mapping) or on the left side (shifted mapping) of the screen. To successfully reach the target, the required hand movement was identical in all conditions. However, the mapping between real and virtual space was changed between normal and shifted viewing conditions. In the normal viewing condition, real space was directly mapped to virtual space. In the shifted viewing condition, shifts of about 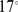 or else 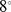 were applied in virtual space, displacing both target position and virtual hand to the left. This emulated the effect of wearing prism glasses which induce a similar shift of a visual scene, while diminishing effects of error correction caused by independent cues about real positions of objects relative to the observer's body.

### Paradigm

The experimental task was the same for all subjects throughout the whole experiment: pointing at a visually presented virtual target. Whenever the subjects pressed the button at the starting position with their pointing hand, a trial began and a pointing target was presented on the screen until the end of the trial. Movements began from this starting position directly in front of the subject's trunk, and ended around the virtual target. Subjects were instructed to move fast but smoothly to the target with no online error corrections, and to try to reach the target with the tip of their right index finger as quickly as possible. They were told that the virtual hand was at exactly the same position as their own hand, and they did not expect any changes in this mapping between real and virtual coordinates. During an initial period, the virtual hand was permanently visible to establish a solid association between the virtual hand and the subject's real hand. During the experiment, proper visual feedback of hand position (virtual hand shown on screen) was provided only at the endpoint of each movement (terminal feedback) for a short time interval (300 ms). The endpoint of a movement was extracted from the recorded trajectory when movement speed fell below a threshold (10 mm/s). After receiving feedback, subjects moved back to the starting position where the next trial started with the button press.

**Figure 2:**
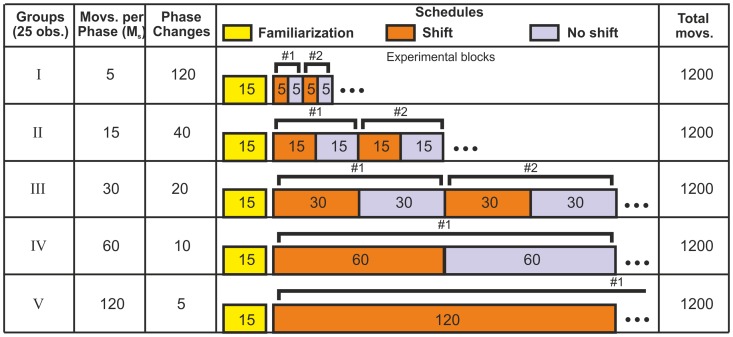
Experimental protocol for dual prism adaptation. Every group was confronted to a different schedule 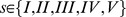 which had a different number of movements per phase 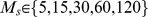, respectively. The total amount of movements was 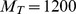, resulting in varying number of blocks 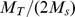 for all different groups 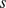. All groups performed 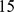 initial setup familiarization movements.

Each subject carried out 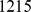 movements in total: 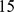 in a familiarization phase prior to the experimental blocks, 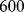 with shifted mapping, and 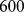 with original mapping. During the familiarization phase, subjects had time to get used to the apparatus and task while pointing in the original mapping. In the subsequent experimental blocks, the different mappings were used in alternating blocks consisting of an adaptation (shifted mapping) and re–adaptation (original mapping) phase each. The number of blocks and phase lengths were varied between experimental groups ranging from 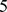 blocks of 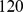 movements each in adaptation and re–adaptation phases, up to 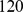 blocks of 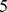 movements in both phases. Figure 2 shows the complete combination of number of blocks and phase lengths. Short breaks were included in the procedure every 300 movements, resulting in three breaks for all groups except for the group with 5 blocks of 120 movements per phase. This group had a break after each block (every 240 movements), resulting in a total of four breaks. Subjects were allowed to stand up and move around during the breaks but stayed within the laboratory room. Each break usually lasted only for a short time (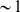 minute), but the subjects were free to continue whenever they felt ready. Each combination of number of blocks and phase lengths was tested in separate groups with both, a large (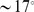) and small (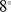) visual shift. In total, ten groups of five subjects each were tested.

### Data analysis

From the pointing trajectories, off–target displacements 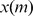 (figure 1), with 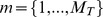 indexing the pointing movements made by each subject were extracted. Displacement was defined as the horizontal distance in degrees of visual angle from the pointing location to the target in the coordinate system of the subject, and corrected by the baseline extracted from the first 15 familiarization trials. Displacement 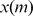 was measured for each observer, labeled with index 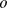, under one of the five experimental schedules 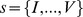 (see figure 2). These three indices are used to unambiguously identify each measured displacement as 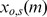. For a small number of movements (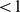
%), the hand speed at the end of the movement did not fall below the threshold for automatic detection. In these cases, pointing error was extracted manually from the pointing trajectory. One of the 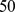 observers did not complete the experiment and was excluded from subsequent analysis.

In order to quantify learning in our dual–adaptation experiment, we focus on the *direct effect*, i.e. the error of the first movements made in every block 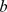 indexed by 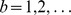. Each block consists of 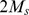 movements, with the first 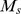 movements made in the shifted mapping and the subsequent 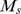 movements made in the original mapping. For each schedule 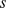, the first movement in block 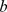 is thus selected by the global movement index 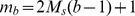. By averaging over the corresponding displacements 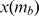, we obtained the average direct effect 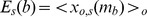. The average *aftereffect* 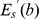, i.e. the mean error of the first movement made after the original mapping was restored, was obtained by averaging displacements with global movement indices 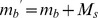.

For a better comparison of data from different schedules, we apply a normalization procedure on average direct effect and aftereffect, which show an exponentially decaying trend with a visible final offset. This dynamics is similar to what is typically seen during single adaptations to horizontal prism shifts [11]. To obtain the normalization, a non–linear least squares fit of an exponential decay plus an offset 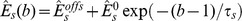 was performed, where 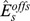 stands for the estimated final offset, and 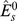 for the estimated direct effect with 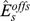 being removed. The final offset is a constant off–target displacement, whose value is typically larger than zero. The decay constant 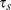 characterizes how fast alternative mappings are learned. The experimental data are normalized by removing the (estimated) offset, and by dividing by the (estimated) direct effect of the first block, 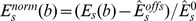. The inverse of the fitted 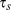 provides an estimate of the learning rate for the acquisition of each mapping during dual prism adaptation. It gives the average number of changes between conditions to reduce the initial off–target displacement by 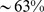.

In order to investigate whether the speed of dual–adaptation is determined by the number of changes between conditions or by the number of executed movements, we analyzed the normalized target deviation 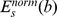 in dependence on executed movements 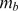. This is done by using the scaling introduced above, where 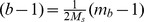, leading to a rescaling of the adaptation constants via 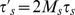. If adaptation speed would depend only on the condition changes, 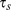 would be constant for all schedules 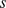. Instead, if adaptation speed would depend only on the number of executed movements, 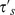 would be constant for all schedules 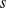.

### Model

**Figure 3:**
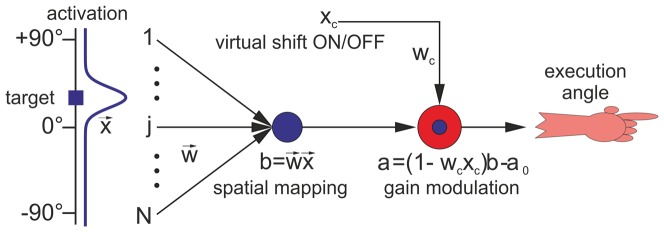
Layout of a perceptron–gain model. The network is able to learn and store dual mappings, acquired during dual prism adaptation with a single target. The Gaussian curve represents input activations coding perceived target location. The spatial mapping is modulated by an adapted gain factor which depends on prism conditions.

As an approach to understand the mechanisms of dual–adaptation, we consider a structurally simple but biophysically plausible adaptation model (see figure 3). Using an essentially one–layered neural network, the model maps a spatially distributed visual input into a reaching direction. Errors between intended and actual hand position are used by a reinforcement algorithm for adapting the parameters of the network, i.e. its synaptic weights. We will first explain the model structure and its mapping from the input to the output variables, and then describe its adaptation/learning dynamics.

### Structure and dynamics

We assume a minimal model with only two stages: An input stage, where a target position is extracted from a spatial activation profile, and an output stage, where the perceived position is transformed into a pointing angle for a motor command. For simplicity, we assume that the spatial activation profile is already in a head–centered coordinate frame, where visual and proprioceptive information (e.g. the actual retinal image and the viewing direction) are already integrated.

#### Input stage

The one–dimensional activation profile 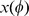 depends on the horizontal angle 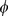. For the dual prism experiment with one target localized at angle 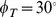, activation is represented by a Gaussian with length scale 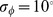,
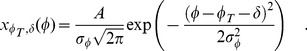
(1)


The variable 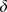 denotes a visual (horizontal) shift induced by wearing prism glasses or by being subjected to a virtual environment. In the normal viewing condition, 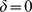, and in the rightwards/leftwards prism condition 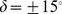. 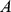 is a free parameter which we have set to 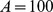. In the following, we assume a discretized profile 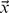 (figure 3) represented in a set of 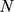 input channels (i.e. neuronal populations). We have chosen the number of input populations to be 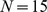, spanning the horizontal viewing angle from 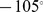 up to 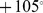. This provides an angular resolution of 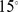, i.e. every input node 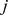 corresponds to an angular location 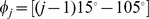, with 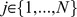. Higher angular resolutions, up to 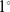 do not affect our main results.

A transformation 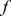 from 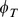 into an encoded perceived target location angle is achieved by computing a scalar product between activation profile 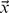 and a positive weight vector 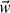. The encoding is denoted by 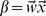, and could be realized by a neuronal population receiving feed–forward inputs from a sensory–integration layer (linear perceptron). By construction, negative neuronal responses do not occur in this model. Assuming that this stage operates in an approximately linear regime, leads us to the expression:
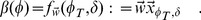
(2)


#### Output stage

The perceived target angle encoded in 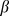 is mapped to a pointing angle 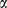 for the execution of the hand movement. For small 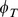 and 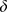 as in the actual experiment, a required mapping 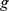 from 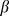 to 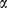 is almost linear (see Discussion and appendix S1), and thus we can write
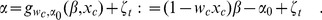
(3)


The parameter 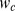 and the variable 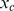 will be explained below. The constant angle 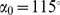 serves to convert the always positive neuronal signal 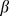 to a pointing angle which can take both positive and negative values. 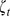 is optionally included as a temporally, statistically independent and zero mean noise source, for a biologically plausible simulation of uncontrolled motor fluctuations and/or uncertainty in neural signaling.

#### Cognitive input

In the dual–adaptation paradigm, the switch between the normal and shifted viewing conditions is accompanied by an apparent change in target position, whose physical location in real space never changes. Subjects might use this indirect information, even prior to experiencing the suddenly large error in their pointing movements, for switching from one learned mapping to a second learned mapping [23], [24]. This cognitive cue processing is here modeled as an adaptive gain mechanism via a second input 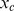, which can be used to instantaneously switch between two gain factors, without requiring to re–learn the spatial mapping embedded in 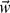. The reason for choosing a multiplicative gain instead of an additive shift will be discussed later. We set 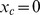 in the normal mapping (prisms OFF), and 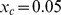 in the shifted mapping (prisms ON). The parameter 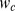 scales the cognitive input and must be learned in order to be able to compensate the visual shift.

#### Dual–adaptation

For a correct pointing movement towards the target, it is required that 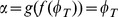. Mathematically, this means that the following two equations must be satisfied, assuming that the target is always presented at 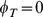:
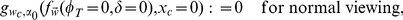
(4)

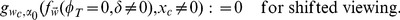
(5)


Using our definitions for 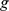 and 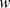, the following equations are obtained:
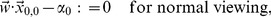
(6)

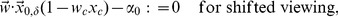
(7)


Equations 6 and 7 are trivially fulfilled for 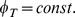, making it possible to acquire a ‘perfect’ dual mapping. However, it will not lead to perfect dual mapping acquisition for target positions 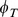 which span the full viewing axis.

### Learning

We propose a reinforcement learning mechanism: A reward or punishment signal is received from interaction with the environment after stochastic exploration of the system's inner parameters has taken place. For instance, adaptation can be achieved by correlating a global signal, which is fed back to the system, with controlled local fluctuations of synaptic connections. The inherent stochasticity of synaptic transmission might serve this purpose [22], [25].

In our paradigm, synaptic fluctuations produce subsequent output fluctuations, e.g. fluctuations of the pointing error. These error fluctuations are taken as the reinforcing global signal. Such a procedure can implement a stochastic gradient learning algorithm [26], thus driving all involved synaptic strengths to an optimum set for realizing the required dual mapping.

The error function is defined as 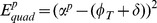, where the supra index 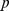 stands for the two possible prism conditions, ON or OFF. When wearing prisms for the first time, the first direct effect will be related to the difference between pointing movement 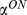 and actual target position 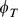 displaced by the visual shift 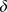. After being adapted to prisms and taking them off, the first aftereffect will be simply related to the difference between pointing movement 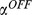 and target location 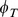. To find an optimum set of synaptic weights, they should be slowly varied towards the direction opposite to the gradient on the error function, as performed by equation 8 (gradient descent),
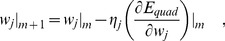
(8)where index 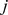 runs along all synaptic weights contained in 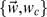, and 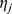 is a small factor to control the learning rate of each synaptic weight. For all ‘spatial’ weights 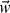, the learning rate is 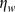, and for the cognitive weight 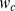, it is 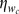. Choosing different values for 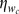 and 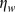 may be required if two distinct neural substrates with different error sensitivities and information acquisition rates are involved [21], [27]. Index 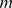 indicates the ordering of the pointing movements as introduced in the Data Analysis section.

The above rule is a theoretical approach to find the optimal weights. A biologically realistic implementation consists in correlating synaptic fluctuations with their impact on the error function, e.g. through some bio–chemical concentrations available to the synapse. Combining these two signals at each synapse allows to estimate the gradient on the error/reward (see [28] for the proof of an alternative mechanism based on the same principles). If a stochastic fluctuation 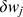 led to a smaller error, a small proportion of it will be expressed as a synaptic change. Instead, if the fluctuation increased error, then a synaptic change in the opposite direction will be taken.

The adaptation procedure is identical for both viewing conditions, which only differ in the values of 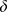 and 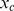 provided as input variables:

- Synaptic weights 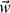 and 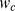 are varied stochastically 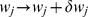 with each fluctuation 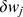 drawn from a Gaussian distribution 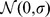, with either 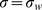 or 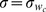 respectively. Since synaptic weights must remain positive, they are clamped to zero if a too large negative fluctuation occurs.
- Synaptic fluctuations lead to output fluctuations 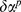, and therefore to error fluctuations 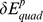. In the experiment, the simultaneously displayed final positions of virtual hand and target are part of this information available to the subject.
- Error fluctuations are fed back to the system to be used as global evaluation signals, i.e. either reward or punishment.
- Correlations between synaptic fluctuations and error fluctuations are used to update synaptic weights, according to a stochastic learning rule
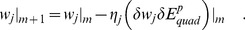
(9)

This learning rule realizes a stochastic gradient descent on the error surface over the space of possible synaptic weight patterns.

Prior to dual–adaptation we let the model learn weights that map each visual stimulation to its correct motor response under normal viewing conditions: We have randomly chosen targets from a uniform distribution over positions in 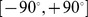 (with above mentioned resolution 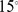) and let the stochastic rule update all synaptic weights. We set 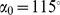. Since 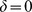 and 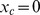, no updates on 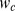 are at this stage relevant for the model's output. Each target was presented for 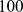 iterations of the learning rule. Learned synaptic weights stabilized after about 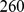 targets.

Weights 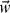 were initialized from a uniform random distribution within 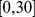 prior to training. After training, we took the average final weights 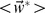 among 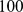 simulations as starting values for the actual dual–adaptation experiment (we also tried non–averaged initializations which could explain some part of observer variability in the real experiment, but of course yielded a higher noise level impairing the subsequent data analysis).

After acquiring normal mapping, a dual–adaptation schedule as in our experiment was realized. When the shifted viewing condition is presented for the first time, the direct effect is ‘perceived’ and fed back to the model. This direct effect must be gradually corrected in order to achieve adaptation. Once adaptation is completed and the normal viewing condition is restored, the model ‘experiences’ an aftereffect. The old mapping must be recovered by a similar adaptation process. In total, the model has two pairs of parameters for controlling the error decay rates, 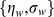 and 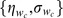.

## Results

### Human observers

**Figure 4:**
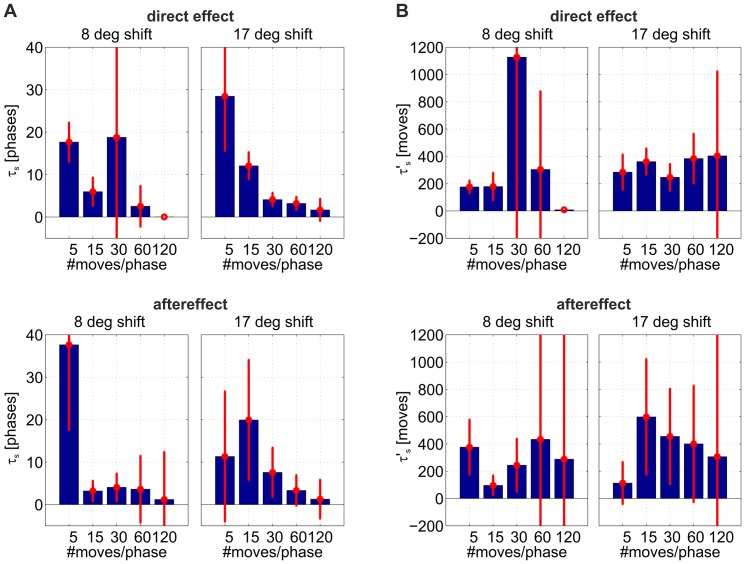
Time constants of exponential fit for the direct effect in adaptation and re–adapation. **A**) Time constants are displayed in units of phase changes. Red lines indicate the confidence intervals (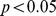) of the exponential fits (due to some very large values, some confidence intervals have been clipped at the graphs' borders). **B**) Same data, now plotted in units of movements.

Pointing errors for both, direct effect and aftereffect, were averaged over observers and fit by an exponentially decaying function with offset (see Methods). Fig. 4 summarizes the results and fits for the time constants 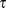 (in units of phase changes, Fig. 4A) and 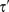 (in units of movements, Fig. 4A). Table 1 and Table 2 summarize the numerical values for the time constants together with the quality of the fit (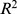) for the small visual shift and large visual shift, respectively. In general, the fits for the small visual shift are not very good – indicating both, a high noise level comparable to the size of the actual visual shift, and the fact that a large proportion of subjects did not adapt very well or even did not perceive this small shift in the virtual environment.

**Table 1:** Time constants for exponential fits for 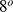 of visual shift.

schedule 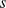	direct effect	aftereffect
5 movements	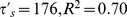	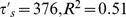
15 movements	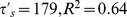	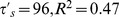
30 movements	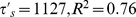	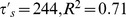
60 movements	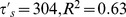	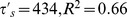
120 movements	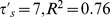	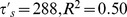

Units for 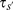 are number of movements.

**Table 2:** Time constants for exponential fits for 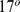 of visual shift.

schedule 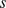	direct effect	aftereffect
5 movements	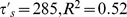	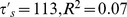
15 movements	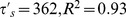	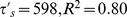
30 movements	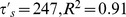	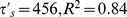
60 movements	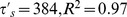	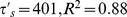
120 movements	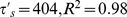	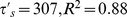

Units for 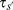 are number of movements.

**Figure 5:**
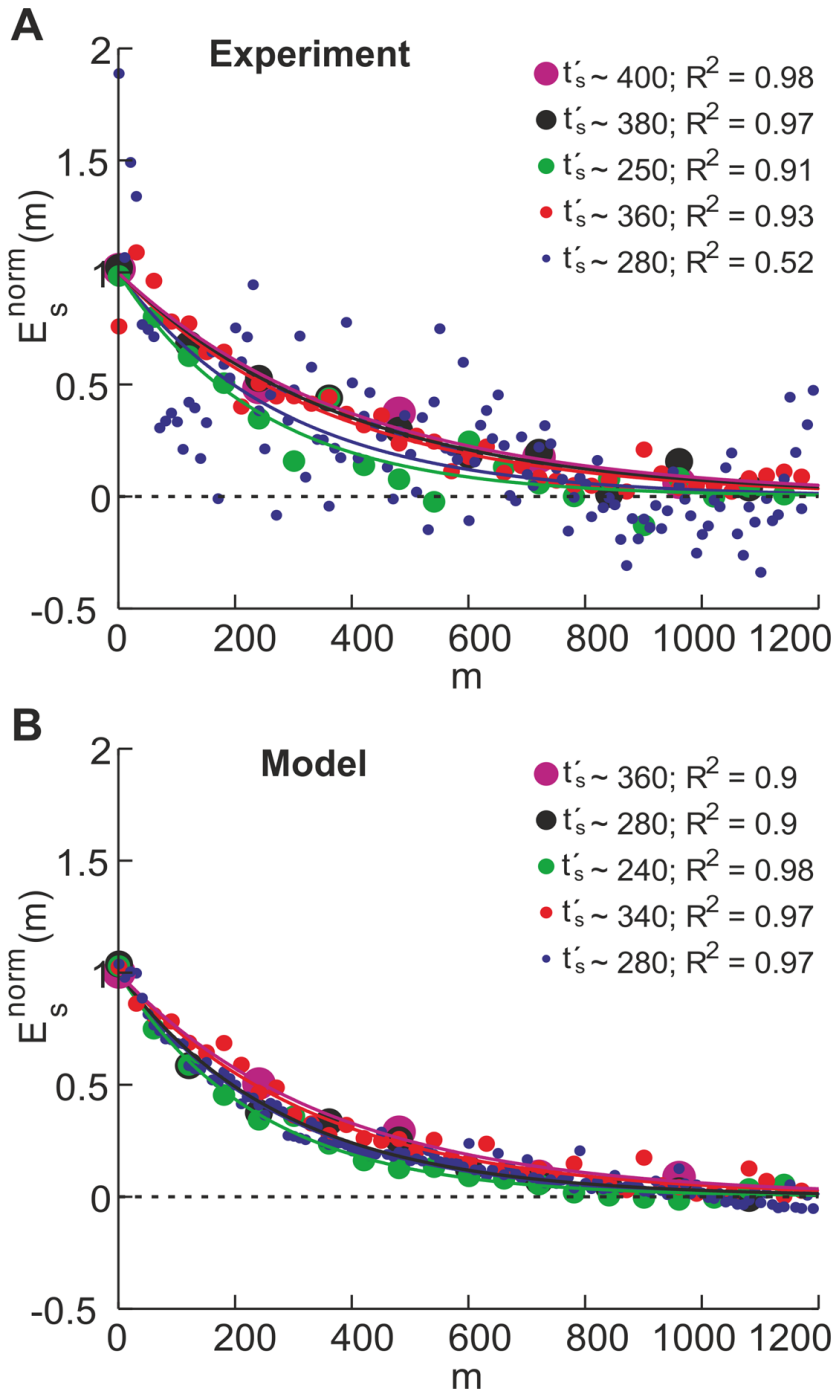
Baselined and normalized direct effect versus index of pointing movement. **A**) **Experiment:** Average out of 5 subjects for each adaptation schedule with left visual shift 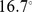. **B**) **Model:** Average out of 100 simulations for each adaptation schedule with right visual shift 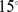.

Focusing on the conditions with large visual shifts (right columns of panels A and B in Fig. 4), it is obvious that 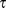 changes much more drastically than 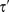. This holds for both, direct effect and aftereffect. Assessment of the goodness–of–fit (red error bars) of each condition reveals no significant difference between the values of 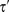. This indicates that adaptation depends mainly on the number of movements made, regardless of their distribution along prism phases. In other words, comparing learning rates for dual–adaptation among experimental schedules reveals no clear evidence of faster acquisition of the dual mapping when changes of prism conditions occur more or less often (see Fig. 5 for the experimental data). When plotting direct effect versus phase changes, the decay rates spread according to the scaling between 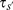 and 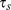 (see Methods). This suggests once more that dual–adaptation depends mainly on the total number of movements, regardless of their distribution along prism phases.

### Model simulations

We performed simulations of the model under similar adaptation schedules as used in the experiment. To reduce variability, 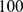 instances of the model were simulated and results averaged over this ensemble. As stated above, the previously learned starting weights 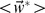 were optimal for the normal mapping. The cognitive weight 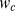 was, however, initialized randomly, from a uniform distribution within 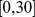. The prism OFF condition was realized by setting 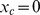 and 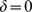, while the prism ON condition was set with values 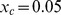 and 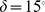.

In the model, each learning step relates to one pointing movement. We used experimental data of only one of the five schedules (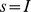) to adjust all parameters to reproduce the learning dynamics (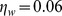, 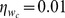, 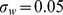 and 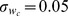). Once fixed, these parameters were also used for simulating adaptation in all other schedules 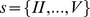. Simulated data were treated identically to experimental ones (see figure 5). As in the experiment, the direct effects from all schedules decay practically with the same rate, depending only on the number of pointing movements.

**Figure 6:**
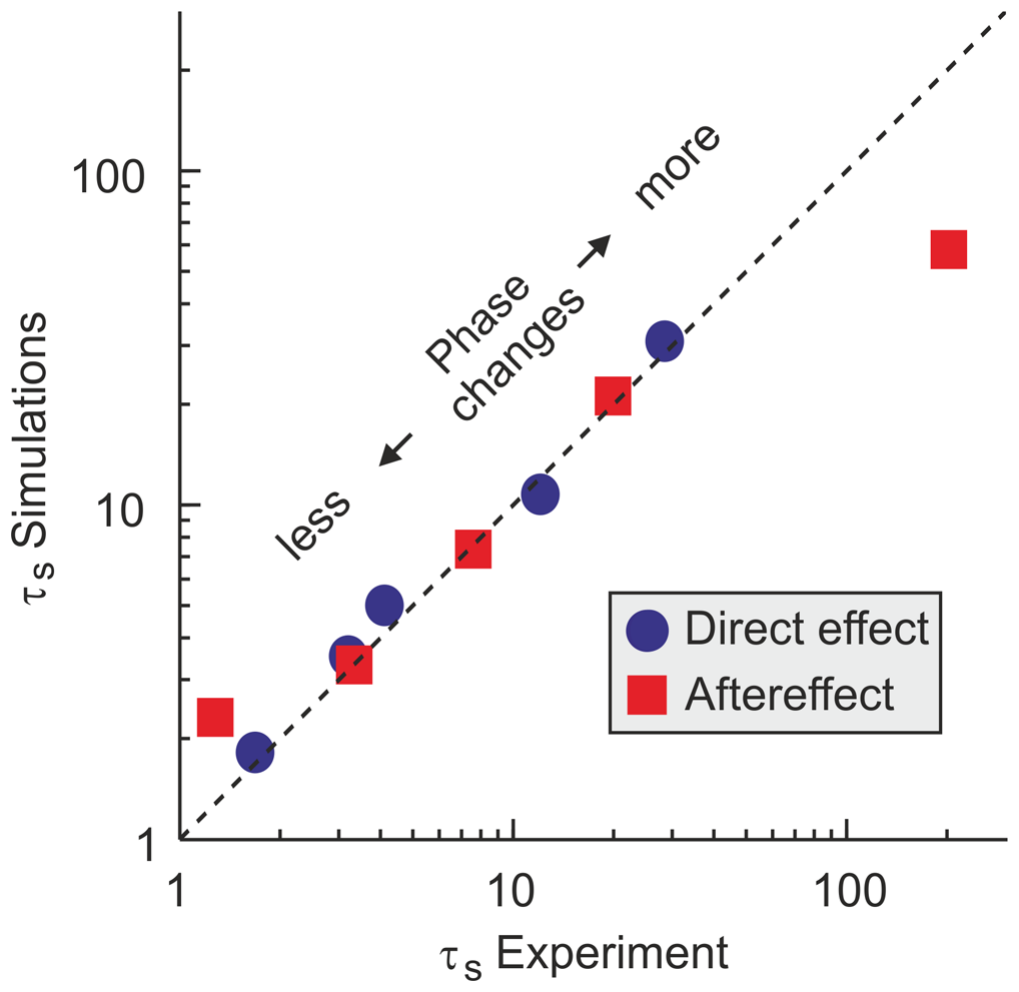
Decay constants 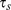 of experimental and simulated direct effect and aftereffects. Values are presented according to their number of phase changes. The diagonal represents perfect agreement between experiment and model predictions.

Aftereffects from experiment and model were also analyzed following our procedure. Figure 6 shows a direct comparison of decay rates among adaptation schedules, according to their number of phase changes. The aftereffect decays slower than the direct effect, and the decay rates also show a dependency on the number of movements only. Again we find a good quantitative match between model and experiment.

The exploration parameters 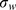 and 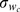 can be varied (within an order of magnitude) around the values given above and still produce similar results for the average dual–adaptation (learning) curves. They influence the fluctuations in the movements of a single simulated subject. In order to achieve learning, the size of these fluctuations must overcome any other non–controlled source of noise, said 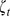. Examples of that would be fatigue of muscles or unreliable neural responses. Once 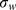 and 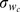 are fixed, learning constants 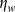 and 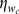 can be chosen to set the time course of adaptation: If 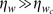, learning affects mainly the stage where a spatial activity distribution is converted into an angle, thus producing a localized adaptation. If 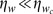, learning mainly changes the slope of the input–output gain function, and thus affects mapping on a global scale, even when adaptation itself takes place only locally. We will focus on this interplay between different adaptation processes in the next paragraphs.

### Spatial transfer and generalization

Spatial transfer and generalization of mappings once adapted on single targets have been extensively investigated [4], [6], [29], [30]. In these studies, subjects typically adapt first to a single target. Subsequently, transfer and generalization of the adaptation are tested by letting subjects point to different target positions for both, direct effect and aftereffect. Results indicate the existence of a rapid local, combined with a slower global process of adaptation. Furthermore, comparing generalization effects for targets left and right to the position of the target used for adaptation reveal spatially asymmetric transfers: The effect is stronger on one side as compared to the opposite side, and depends on the direction of the applied visual shift [31], [32].

Spatial transfer and generalization can also be analyzed in our model, in which local transfer of adaptation occurs due to synaptic changes in 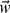, and global transfer due to changes in 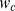. At any point in time during adaptation, the learned synaptic weights can be used to predict pointing errors to different target positions left or right from the adaptation target.

**Figure 7:**
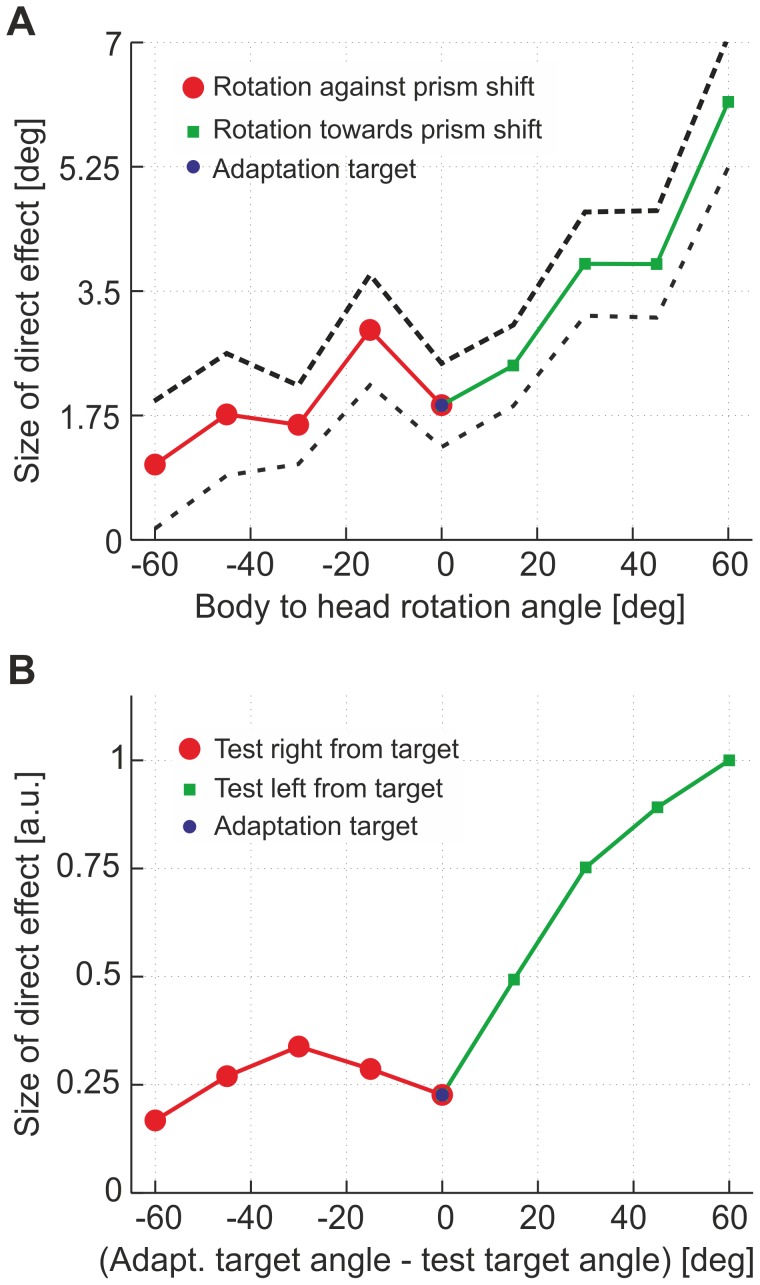
Spatial transfer of adaptation. **A**) **Experiment:** After single target adaptation, the direct effect was tested for different body to head rotations (after [31], [32]). Data include left and right shifting prisms. Rotations towards prism visual shift led to a monotonously increasing direct effect, and against prism shift to a shallower trend with a light bump. **B**) **Model:** Simulation of single target adaptation (right shift). The direct effect is tested rightwards and leftwards from adapting target. Testing rightwards from adapting target is equivalent to testing at the same target once the body is rotated leftwards. Asymmetric spatial transfer of the direct effect is commonly observed in experiment and model.

We compared model predictions to data from the experiment by Bornschlegl and Wischhusen [31], [32], where first adaptation to a shifted viewing condition was performed by pointing to a fixed target position. Subsequently, generalization of the adaptation was tested by rotating the subject's trunk clockwise or anti-clockwise and recording the resulting pointing error. In our model, this setup is equivalent to adapting to a single target and measuring the direct effect by pointing to test targets leftwards or rightwards from that adapted target. Specifically, every leftward/rightward body rotation configuration in the above mentioned experiment can be represented by a testing target right/left from the training target in our model (figure 7). It turns out that the direct effect has a local minimum (dip) at the trained position, superimposed on a global scaling reducing the direct effect for negative angles, and increasing it for positive angles.

**Figure 8:**
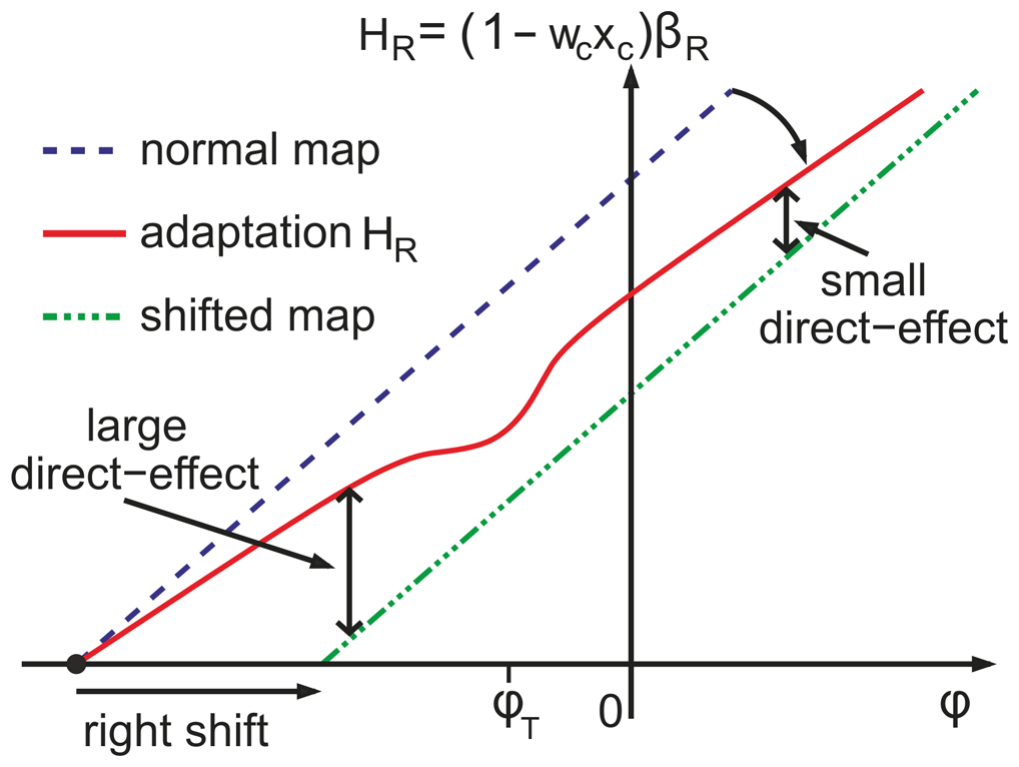
Spatial transfer of adaptation. Around 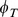, the normal map is enabled when 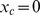. Adaptation 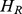 matches the shifted map at the same target when 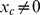. On the side contrary to visual shift (head rotation against it or trunk rotation towards it), the direct effect is larger than on the other side, since error corrections made by the gain factor are smaller.

The width of the local dip in figure 7 (B) is determined by the scale 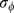 of the input activation, which becomes imprinted in the spatial weights 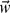 when adaptation at a single target position takes place (see also scheme 8 for a graphical explanation). Outside this range, the direct effect becomes larger and makes place for a global gain modulation (changes in 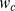). By definition, this gain adaptation causes larger pointing errors on one side of the ‘gaze–motor mapping’ (left side in figure 8), and smaller pointing errors on the other side (right side in figure 8). Thus, the direct effect is enhanced on the side contrary to prism shift (or as in the mentioned experiment, body rotations towards prism shift), and suppressed on the other side (for extension of this result to an agonist/antagonist scheme, see figure 11, and the explanations in appendix S1). The combination of these local and global adjustments results in an asymmetric transfer, as observed in prism adaptation paradigms with human subjects [6], [31]–[33]. Generalization of prism adaptation is described in [6] where recalibration (remapping of spatially coded movement to rapidly reduce error) and realignment (transformation of spatial maps to bring origins of coordinate systems into correspondence) are proposed [21]. In our model the local and global transfer of adaptation could resemble, at least on a short time scale, those recalibration and realignment mechanisms.

## Discussion

In summary, we investigated dual prism–adaptation in a single target pointing task by combining experiments with neural modelling. The experiments revealed that subjects adapt to the two environments with an exponentially decaying error in the direct effect and aftereffect. This decay mainly depended on the number of movements made, and was only weakly influenced by the learning schedule, i.e. the number of phase changes during a fixed number of movements. The model quantitatively reproduced the experimentally observed dynamics (i.e. the learning curves) of both, direct effect and aftereffect. Acquisition of dual mappings resulted independently of the particular learning schedule. In our model, dual–adaptation is a process based on reinforcement learning. The error decay constant of this process only weakly depends on initial conditions, while mainly on the parameters 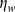, 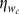, 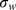 and 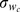. This implies that the sequence of learning steps performed under both prism conditions is irrelevant for the final result. In consequence, also the decay constants of direct effect and aftereffect inherit this property and do not depend on the particular learning schedules, thus providing an explanation for our main experimental result.

The combination of a local and a global adaptation mechanism in the model explains spatial generalization of prism adaptation as observed in independent experiments in a qualitative manner. Taken together, our model provides a unifying, neurally plausible approach for adaptation in a variety of settings. In the following, we will discuss extents and limitations of our model.

### Dual–adaptation learning speed

A previous work reports different decay rates of the pointing error during prism adaptation, reflecting different available sources of information [34]. On the basis of our results, we argue that at least the information about phase change is largely irrelevant for the average decay speed of the pointing error during dual–adaptation. Instead, we recognize the pointing movement with terminal visual feedback as the main regulator of dual–adaptation. This suggests that learning proceeds practically without loss between periods of prism wearing, and achievements are preserved over the breaks between prism sessions. Learned skills can be stored over long periods of inactivity. This means: at least some new sensori–motor maps are acquired by practicing them in any order, and they are not easily unlearned [2].

In addition, we find that in the model, learning speed after the first movement in one condition is constant and does not increase with the number of phase changes. This result is consistent with the behaviour of more abstract ‘state–space’ models [5], [35], [36]. From our experimental data, we were not able to determine if learning speed within a phase increases or stays constant over the sequence of phase changes, because the noise level turned out to be too high.

It has also been observed that random schedules, i.e. unpredictable changes between prism conditions, improve consolidation and retention of the different mappings (e.g., [23]). In our experiment, the changes were predictable, at least in those conditions with a large number of phase changes. However, many subjects did not report having consciously perceived the changes between conditions when they were questioned after the experiment. Therefore we expect that our results generalize well to situations with random changes.

In the model, we also tested how learning speed depends on uncertainty/noise in representation of the target (parameter 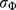). If 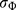 was increased, we observed also an increase in the adaptation time constant 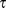. This finding is consistent with experimental data [18]. As 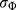 determines also the width of local spatial generalization, the model thus predicts a larger range of the local transfer if the representation of the target is subject to a larger amount of noise.

Adaptation takes also much longer when the model learns to represent two shifted conditions (e.g., to +7.5 and −7.5 degrees shifts) as compared to learning one ‘normal’ and one shifted condition of the same absolute angle difference (e.g., to 0 and +15 degrees shifts).

### Subject variability and fitting biases

Our analysis of dual–adaptation was focused on the average learning speed in model and experiment under particular adaptation schedules. In our experiment, initial amplitudes of pointing error, adaptation rates and final offsets varied from subject to subject. In contrast, no statistically significant variation of noise level among the different scheduled groups can be observed.

The learning speed is identical for all instantiations of the model (i.e., initialization of weights), but single simulations display much noise and need averaging for a proper estimation of learning rates. The noise level becomes important when estimating adaptation rates for different training schedules, which provide different amounts of data points to be fitted (shorter period lengths provide fewer data points per period). Especially when adaptation is still not complete after performing all movements of each period, there is a systematic bias towards slower decay rates for fewer available data points, because it is more difficult to estimate the final offset. This trend is seen in the experimental data, and confirmed by tests with surrogate data (not shown).

### Direct effect versus aftereffect

One observation in dual prism adaptation is the difference in decay rate between direct effect and aftereffect. Data from our experiment, as well as from other studies [2], [11], show that the aftereffect decays slower than the direct effect. Furthermore, while manipulating experimental conditions during the adaptation to the prism ON condition drastically modulates the dynamics of the direct effect, the aftereffect remains unaffected by those manipulations [23], [37]. A possible explanation of these findings is that certain mechanisms, which are enabled during adaptation in the prism ON condition, might not take place during adaptation in the prism OFF condition. This is the case in our model, where during re–adaptation phases, the cognitive input is disabled (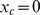). Thus changes in the cognitive weight 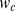 become irrelevant for the system's output (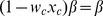) and fluctuations in 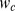 decorrelate with the reinforcement signal. In consequence, re–adaptation exploits only spatial weight changes 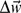, while prism ON adaptation exploits changes both in 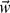 and 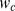, thus speeding up learning and reproducing the experimental observations (figure 6).

### Size of first pointing error

In most prism adaptation experiments, the initial size of pointing errors is clearly smaller than the actual prism shift [12], [38]–[40]. Non–controlled independent cues have been proposed as the cause of this effect. For example, geometrical hints in rectangular rooms, alignments of the experimental setup and a strong prior of an artificially induced visual shift can indicate the real target location and counteract its optical shift. However, it has been found that this effect is also present in virtual reality setups [18], where suppression of those independent cues is much easier. In our experiment, the change from the non–shifted to the shifted mapping could be inferred by the subjects from the accompanying shift of the target and shift of the frame within their visual field.

In our model, the size of the initial pointing error depends only on the mean value of the randomly initialized cognitive weight 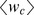. Specifically, if 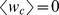, the initial direct effect equals the induced visual shift. If 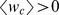, the initial direct effect becomes smaller, as observed in humans. Such a non–zero 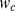 can be interpreted as a prior on the magnitude of a compensatory input necessary to counteract a visual shift. Hence, this free parameter 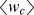 can be set to reproduce this feature from the initial direct and aftereffect. Since humans must adapt to environmental perturbations every time and at anytime, having a prior gain ‘ready’ for cognitive or contextual cues about a perturbation might prove useful. It improves the response to large perturbations by starting with an initial correction, hence reducing learning effort and time.

### Model limitations and extensions

#### First stage: Spatial mapping

In our experiment and in [31], [32], the retinal image is almost independent of the experimental condition, as subjects always fixate the target before performing a pointing movement. In our model, however, we provide a head– (or body–) centered representation of visual space as input to the first stage. We assume that this representation is not directly derived from retinal activation, but occurs at a later cortical stage, where visual inputs (retinal images) along with proprioceptive signaling (eye muscles) are already integrated [17], [41]. There is some evidence for such ‘gain fields’ in certain brain areas (for an overview, see [42]). It is straightforward to extend the model and to incorporate such a stage; the most simple realization would be to assume a spatially distributed visual input as the one on the retina from which a target angle is derived, and then a stage where proprioceptive feedback is integrated by e.g. removing the eye position from the computed angle. It is also easy to extend the model to cover adaptation in other actuators (see explanations in appendix S1, and the figure S2).

The resolution of the spatial representation can be refined up to 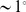. Learning steps and learning parameters must then be re–configured to achieve similar results. To reproduce the experimental data, it is not necessary to perform exactly one learning step after one movement. Specifically, 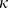 learning steps per movement can be performed, as long as 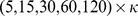 iterations of the learning rule are performed per prism condition for the different schedules. The absolute scale 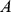 of the spatial representation is arbitrary, as it becomes normalized by the mean of the synaptic weights, which is adjusted by the learning procedure.

#### Second stage: Gain mechanism

Instead of a gain mechanism, a global additive shifting would also solve the local dual–adaptation problem, transfer the shift to the surrounding locations, and thus provide a perfect generalization. The reason to chose the, at first sight, much more inappropriate gain mechanism was motivated by the observation of an asymmetric spatial transfer, when humans adapted to a single target. We indeed found that a global shift can account for the identical decay constants in all schedules, but can not explain the asymmetric spatial transfer (data not shown).

For simplicity, we neglected the size of the human body and assumed that a change 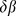 in target position would require an equal change 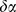 in pointing direction. This is approximately correct if the ratio of head's pivot to shoulder distance over arm's length is relatively small (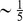). For all angles of the perceived target we have that 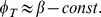, and so, the required hand movement angle 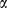 will approximately linearly depend on 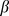. However, there exists a non–linear correction with an upper bound of 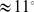 due to the mentioned ratio of 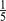 (see appendix S1 for the derivation, and figure S1 for illustration). Due to the noise in the experimental data, deviations of the model caused by these geometric factors are difficult to investigate.

More importantly, experimental evidence from [31], [32] might indicate the existence of both, agonist and antagonist mechanisms for adjusting the output gain. Depending on the direction of the visual shift, only one of these parts is subject to adaptation, at least on time scales relevant for the situations explained by our model. However, for every particular subject, the visual shift during the experiment was always into the same direction. No subject was exposed to different visual shifts. Therefore, we could simplify this situation by incorporating only one gain factor, hereby avoiding to introduce a further control mechanism for differentially activating learning of these two gain factors depending on the situation (see also appendix S1 and the illustration in figure S3).

Finally, we must point out the simplicity of the global gain adaptation implemented in our model. The question about the range of spatial transfer and generalization is still under discussion [5], [43]. A possible refinement of our model would be to implement a less global adaptive gain mechanism 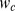, still with a wider effective range than adaptation on the spatial mapping 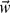, whose length scale is determined by 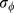.

## Supporting Information

Appendix S1 **Additional information for the model setup and generalization of results.**
(PDF)

Figure S1 **Top–down perspective of the head, body, eyes, prisms, arm and target configuration.** A sketch of the proposed reference frame to measure and relate all involved angles and lengths during target fixation and pointing movements.
(TIF)

Figure S2 **Flowchart of the combination of angular variables for target, prisms and body parts.** Adaptation individually modifies the execution of a trained actuator, while other untrained actuators are minimally affected.
(TIF)

Figure S3
**Spatial transfer of adaptation.** The antagonist version 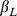 of the mapping 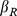 presented in the main text is here depicted. If modeling left visual shifts, the normal left map would be a reflection of the right one. However, it would be adapted with a similar gain modulation 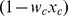. As in the agonist version, around 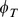, the normal map is enabled when 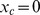. Adaptation 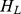 matches the shifted map at the same target when 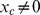. On the side contrary to visual shift (head rotation against it or trunk rotation towards it), the direct effect is larger than on the other side, as observed in experimental data [31], [32].(TIF)
