# Supplementary material for: Dynamics of Dual Prism Adaptation: Relating Novel Experimental Results to a Minimalistic Neural Model
Source: PLoS One. 2013 Oct 29;8(10):e76601. doi: 10.1371/journal.pone.0076601 (PMC3812208; doi:10.1371/journal.pone.0076601)
Supplement: Appendix S1 — Additional information for the model setup and generalization of results. (PDF) [file pone.0076601.s001.pdf]

Appendix S1

Fitting method

The fits of each curve was computed with a non–linear least squared errors (NL–LSE) fitting method, which is basically an iterative fitting algorithm. This algorithm was chosen because of the following mathematical reasons: The model of the data consists of an exponential function plus a non–zero offset, and this causes the simple least squared errors method (LSE) to be not appropriate. The LSE method leads to derivatives that depend on the independent measurements as well as on the model's parameters in a non–linear fashion. In order to find the values of the model's parameters, the obtained system of equations with the LSE method requests to solve transcendental functions. An example is the case of having to solve for λ given the equality $\exp(C_1\lambda) = C_2\lambda$, with C_1 and C_2 as constants. No analytical isolation of the variable λ is here possible. Nevertheless, an initial value close to a guessed correct value from λ can be provided to the NL–LSE method. Then, the correct value of λ can be found, up to a certain error tolerance, thanks to iterative forms given by the NL–LSE method. In summary, the parameters of all here fitted exponential curves with offset are found numerically after a large enough number of iterations of the NL–LSE formulas.

Goodness of fit

The goodness of fit was evaluated through its coefficient of determination R^2. For this, the residual sum of squares $S_{err} = \sum_{\nu=1}^{N}(X_\nu - \hat{X}_\nu)^2$ and the total sum of squares $S_{tot} = \sum_{\nu=1}^{N}(X_\nu - \bar{X}_\nu)^2$ must be computed. Here, X_ν is the νth measured data point, $\hat{X}_\nu$ is its estimation coming from the fitting model and $\bar{X}_\nu$ is the average of all data points $\frac{\sum_{\nu=1}^{N} X_\nu}{N}$. The coefficient of determination is calculated according to the definition $R^2 \equiv \left(1 - \frac{S_{err}}{S_{tot}}\right)$. The value $R^2 = 1$ represents the best goodness of a fit achievable by an arbitrary model. In all prism adaptation data sets analyzed in this paper, the model chosen to fit them was an exponential decay plus an offset. In all plots presenting a fit of an exponential decay, the fitted data are shown accompanied by their corresponding coefficient of determination.

Reference frame

A reference frame is here defined, in which the relationships among all angles and body lengths involved in the execution of a pointing movement are expressed. This reference frame is fixed to the subject's head with its origin at the pivot point P. See figure S1 for identification of all angles and lengths. All rotation angles are defined around vertical axes, where clockwise rotations are taken as positive, and counterclockwise as negative. We describe trunk rotations with the angle θ, and eyes rotations with the angle ϕ. Since $\overline{lr} \ll \overline{AT}$, we assume that both eyes possess the same angular orientation when looking towards the target. The angle γ describes the direction towards the target T with respect to the eyes, being target fixation at $\gamma = 0$. The angle δ represents the visual shift of the target T to a perceived position $\hat{T}$. The shift is induced by wearing prisms, or by an homogeneous horizontal shift in the virtual environment. Finally, the angles α and $\hat{\alpha}$ are arm's pointing angles without and with visual shift respectively.

We denote the distance from head's pivot to the middle point between eyes as $\overline{Plr}$. We also define the angle from head's pivot to target, when wearing prisms, as $\xi_{\hat{T}}$. According to the sine law, to the sum of internal angles of a triangle and to the trigonometric identity $\sin(a \pm b) = \sin(a)\cos(b) \pm \cos(a)\sin(b)$, the angle $\xi_{\hat{T}}$ is computed:

$$\xi_{\hat{T}} = \operatorname{arc}\cot\left(\cot(\phi+\gamma+\delta) + \frac{\overline{Plr}}{\overline{P\hat{T}}\sin(\phi+\gamma+\delta)}\right) \quad . \tag{1}$$

The distance from head's pivot to apparent target $\overline{P\hat{T}}$ needs not to be known. In fact, $\overline{P\hat{T}} \gg \overline{Plr}$, and so, equation 1 is approximated to $\xi_{\hat{T}} \approx \phi + \gamma + \delta$. By using the same trigonometric methods and identities, the expression for the pointing angle as a function of all other angles and lengths $\hat{\alpha}\left(\phi, \gamma, \delta, \theta, \overline{PA}, \overline{A\hat{T}}\right)$ is obtained:

$$\hat{\alpha} = \phi + \gamma + \delta - \theta - \arcsin\left(\frac{\overline{PA}}{\overline{A\hat{T}}}\cos(\theta - \phi - \gamma - \delta)\right) \quad . \tag{2}$$

The ratio $\frac{\overline{PA}}{\overline{A\hat{T}}}$ can not be neglected. It represents a proportion of length from head's pivot to shoulder over arm's length ($\sim \frac{1}{5}$). Nevertheless, the cosine has a bounded magnitude of 1. The maximum magnitude $\arcsin\left(\frac{\overline{PA}}{\overline{A\hat{T}}}\right)$ will represent an error of ($\arcsin\left(\frac{1}{5}\right) \approx$

11°) between seeing and pointing towards the center. Relationships between target location and execution command are approximated as it follows:

$$\hat{\alpha} = \phi + \gamma + \delta - \theta - \alpha_0' \quad (3)$$

$$\alpha = \phi + \gamma - \theta - \alpha_0' \quad . \quad (4)$$

With these approximations, the perceived target angle ϕ mapped to a pointing angle α for the execution of the hand movement is taken as linear. In the model, the perceived location is encoded through a neural activation $\beta = \vec{w}\vec{x}$, such that $\phi_T \approx \beta - const$. All these considerations justify the formulation of the model, which expresses $\alpha = (1 - w_c x_c)\beta - \alpha_0$, since equations 3 and 4 show the similar linear form. Prism adaptation has proved to be mostly specific to the trained body part. Transfer of adaptation to actuators, or motor patterns, different than those exposed to training results very low or null in the proprioceptive system [1]. Thus, a flowchart of the here proposed involved stages is shown in figure S2.

Spatial transfer and generalization

Two adapting mechanisms, a local one at weights $\vec{w}$, and a global one at the gain w_c, cause asymmetric spatial transfer of adaptation. Learning occurring at weights $\vec{w}$ leads a to rapid correction of pointing errors at a single training target, once a visual shift was induced. However, another mechanism, probably slower and with a broader spatial influence, provides also a strategy to correct pointing errors. Figures 8 (in the main manuscript) and S3 show how these two processes account together for asymmetric spatial transfer. The normal map is a codding β (or β_R for the right shift case) of the perceived target location ϕ_T. In presence of a right/left visual shift, the target location codding for an agonist/antagonist side must adapt. If it is desired to keep the former mapping from normal condition unaffected, the gain factor $(1 - w_c x_c)$ must accomplish this around the training target. The size of the direct effect, when testing it at targets around the training one, is the difference between the shifted map and the, until that moment, adapted map.

Due to the gain modulation, the direct effect on one side of the training target is smaller than on the other.

In order to have a more robust codding of the perceived target, a combination of agonist H_R and antagonist H_L mappings could be implemented. This results in a refinement of the model that, in the pursuit of simplicity, was not presented in the main content. For instance, the linear combination $\alpha \propto (H_R - H_L)$ (figures 8 (in the main manuscript) and S3) would represent a coding from perceived target location up to angle of command execution. By construction, this would span negative and positive angles of execution movements. After our experimental and modeling studies, it is still under discussion whether prism adaptation can be consistent with a gain modulation present in only one of the two systems (agonist/antagonist) at a time, in dependence on the induced prism shift.

References

1. Harris CS (1965) Perceptual adaptation to inverted, reversed, and displaced vision. Psychological Review 72(6): 419–444.
