# Supplementary figures and images for: Dynamics of Dual Prism Adaptation: Relating Novel Experimental Results to a Minimalistic Neural Model
Source: PLoS One. 2013 Oct 29;8(10):e76601. doi: 10.1371/journal.pone.0076601 (PMC3812208; doi:10.1371/journal.pone.0076601)

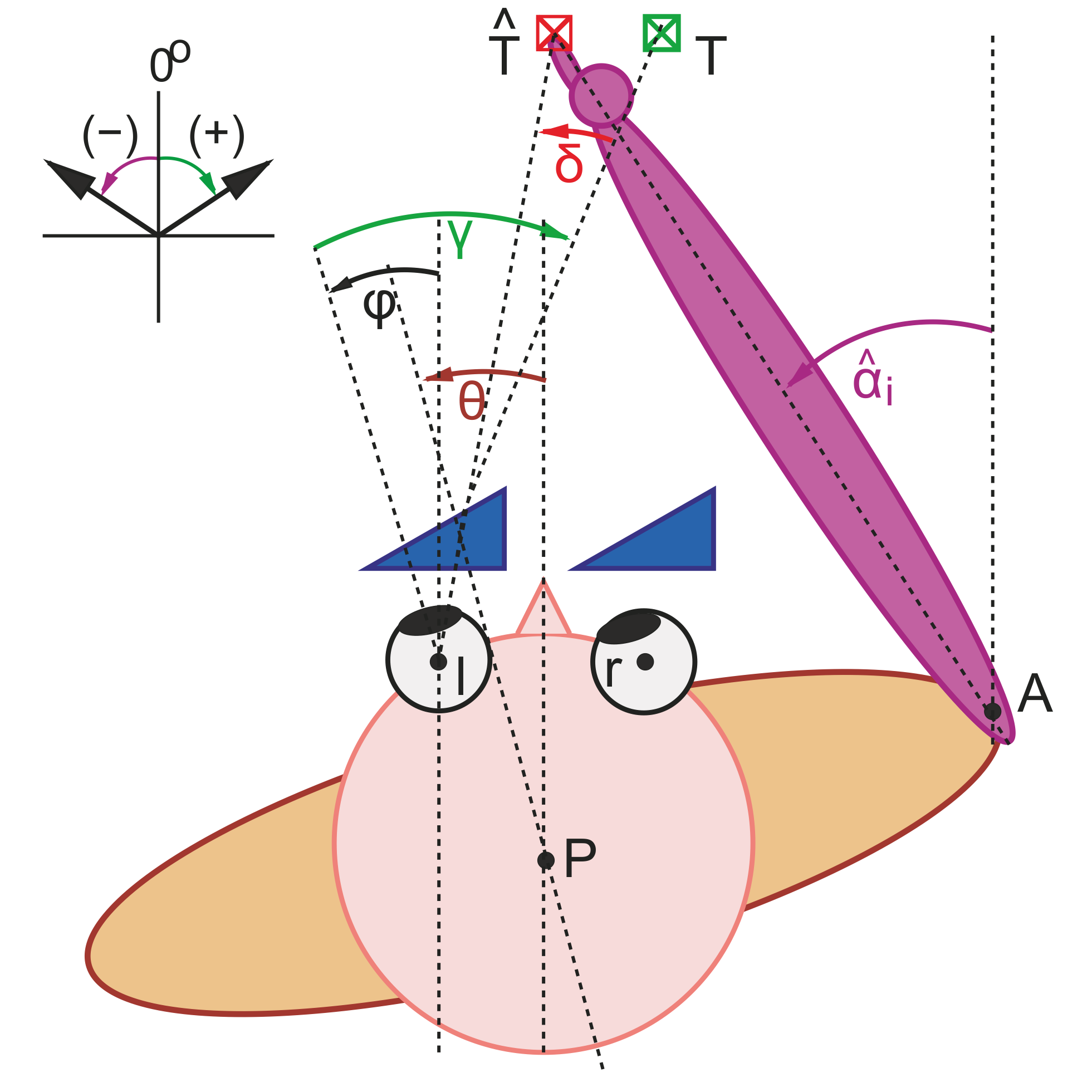

Supplement: Figure S1 — Top–down perspective of the head, body, eyes, prisms, arm and target configuration. A sketch of the proposed reference frame to measure and relate all involved angles and lengths during target fixation and pointing movements. (TIF) [file pone.0076601.s002.tif]

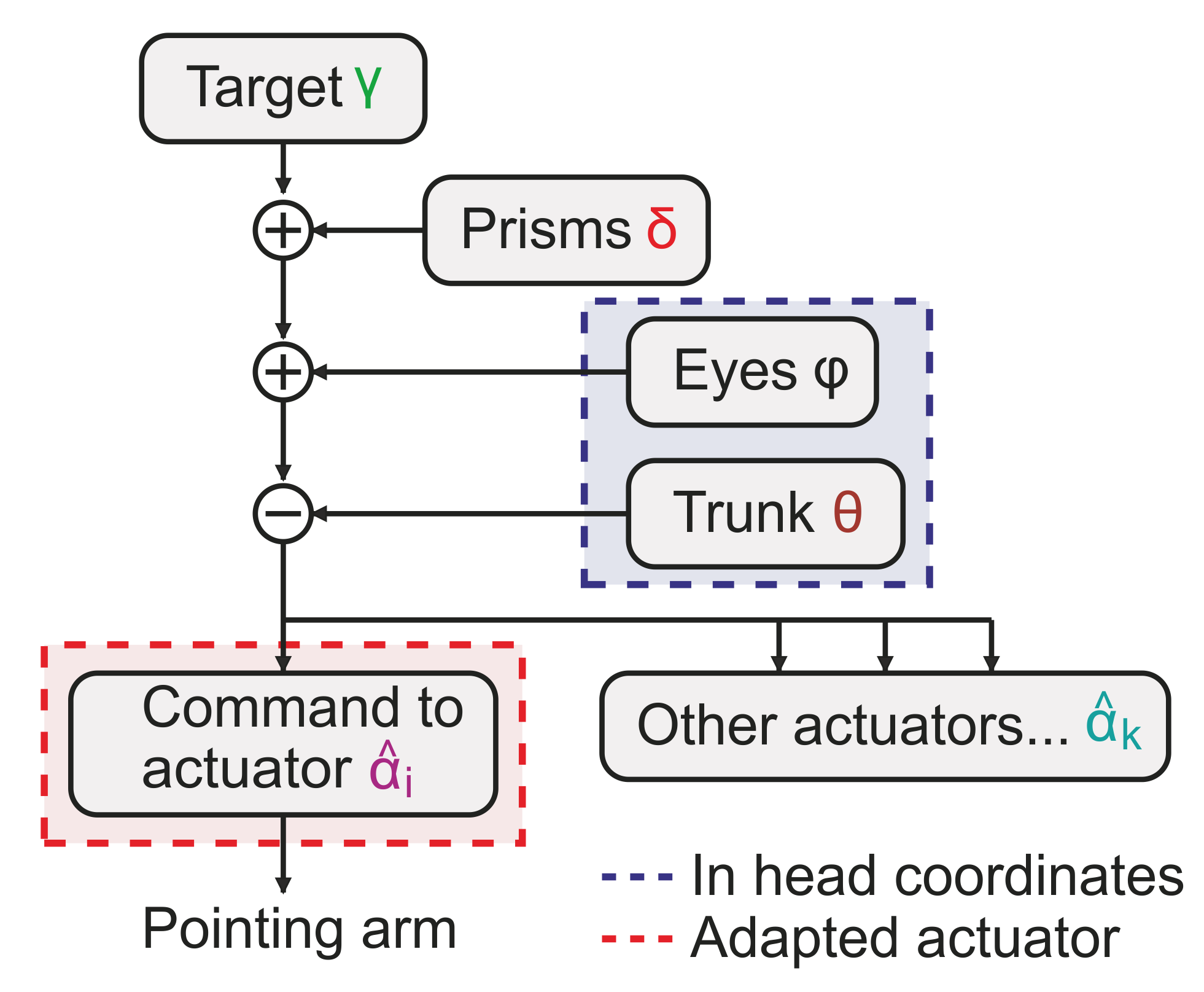

Supplement: Figure S2 — Flowchart of the combination of angular variables for target, prisms and body parts. Adaptation individually modifies the execution of a trained actuator, while other untrained actuators are minimally affected. (TIF) [file pone.0076601.s003.tif]

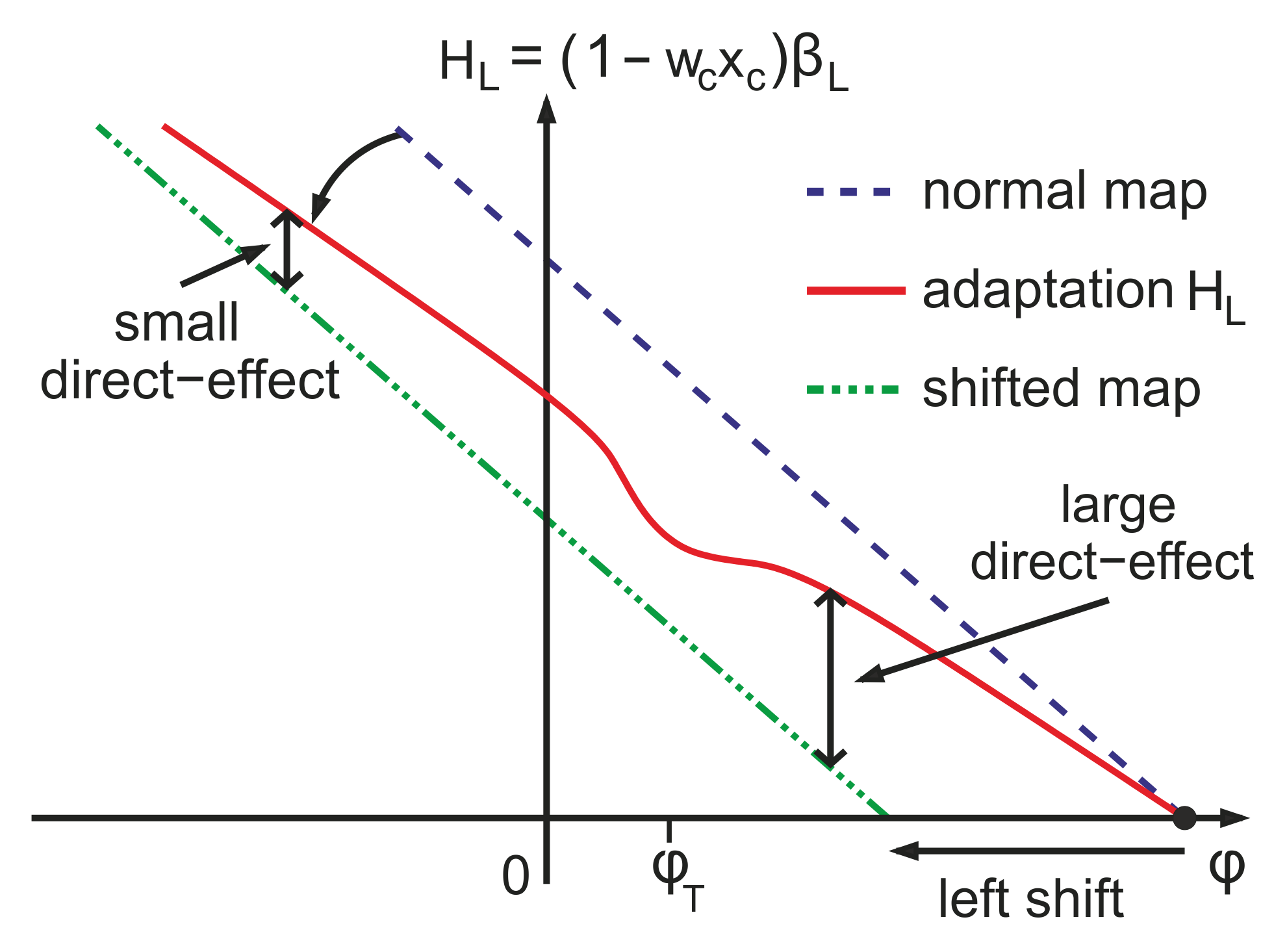

Supplement: Figure S3 — Spatial transfer of adaptation. The antagonist version of the mapping presented in the main text is here depicted. If modeling left visual shifts, the normal left map would be a reflection of the right one. However, it would be adapted with a similar gain modulation . As in the agonist version, around , the normal map is enabled when . Adaptation matches the shifted map at the same target when . On the side contrary to visual shift (head rotation against it or trunk rotation towards it), the direct effect is larger than on the other side, as observed in experimental data [31], [32]. (TIF) [file pone.0076601.s004.tif]
